# In-silico design, synthesis, ADMET studies and biological evaluation of novel derivatives of Chlorogenic acid against Urease protein and *H. Pylori* bacterium

**DOI:** 10.1186/s13065-019-0556-0

**Published:** 2019-03-28

**Authors:** Ritu Kataria, Anurag Khatkar

**Affiliations:** 1 International Institute of Pharmaceutical Sciences, Sonepat, Haryana India; 2 Laboratory for Preservation Technology and Enzyme Inhibition Studies, Faculty of Pharmaceutical Sciences, Maharshi Dayanand University, Rohtak, Haryana India

**Keywords:** Chlorogenic acid, Antioxidant, Urease inhibition, Anti- *H. pylori*, Polyphenolic

## Abstract

**Background:**

Plants have always played important role in treating human and animal diseases as a therapeutic agent for traditional medicine. Through extensive research throughout the world, potential of natural products have been identified to control the over activity of many enzymes. *In*-*silico* screening a library of chlorogenic acid derivatives highlighted some novel compounds which were found effective against urease enzyme and cancer causing *H. Pylori* bacterium. Selected top ligands possessing minimum binding energy and good docking score were synthesized in wet lab by suitable procedure and evaluated for urease enzyme inhibition and free radical scavenging property. Synthetic scheme includes three step reactions i. e protection of hydroxyl group of quinic acid part of chlorogenic acid with lactonisation process, anilide formation by reaction with substituted anilines followed by extraction with ethyl acetate under vacuum and deprotection of hydroxyl groups by treatment with hydrochloric acid.

**Results:**

In-vitro results of the series concluded that compounds **C4a**, **C4d** and **C4b** (IC_50_ 11.01 ± 0.013, 13.8 ± 0.041 and 15.86 ± 0.004 µM respectively in urease inhibition and 5.10 ± 0.018, 5.34 ± 0.007 and 6.01 ± 0.005 µM in antioxidant property against DPPH) were found to be significantly potent with excellent dock score − 10.091, − 10.603, − 9.833 and binding energy − 62.674, − 63.352, 56.267 kg/mol as compared to standard drugs thiourea and acetohydroxamic acid (− 3.459, − 3.049 and − 21.156 kJ/mol and − 17.454 kJ/mol) whereas compounds **C4c**, **C4**(**e**, **h**) exhibited moderate in vivo activity when compared to standard.

**Conclusions:**

Selected candidates from the outcome of in vitro urease inhibitory were further examined for anti-*H. Pylori* activity by well diffusion method against *H. pylori* bacterium (DSM 4867). Compound **C4a** showed significant anti-*H. Pylori* activity with zone of inhibition 10.00 ± 0.00 mm and MIC value 500 μg/mL as compared to standard drug acetohydroxamic acid having zone of inhibition 9.00 ± 0.50 mm and MIC 1000 μg/mL. Molecular docking studies also showed that compounds show strong inhibition by forming strong hydrogen bonding interactions with residues of pocket site in target protein. Hence, the present investigation studies will provide a new vision for the discovery of potent agents against *H. Pylori* and urease associated diseases.

## Introduction

Enzyme inhibition studies has been the interesting topic of research today since a large number of diseases are directly or indirectly associated with some enzyme. A hydrolytic enzyme, urease is found abundantly in nature in soil as well as in a large number of microbes like bacteria, algae, fungi. Urease is beneficial for plants as it provides nitrogen to them which is essential for their growth but its excess activity may harm environment by producing excess of ammonia. In human beings it serves the role of a pathogen and responsible for many serious disease like gastric cancer, peptic ulcer, crust formation in catheter, hepatic coma, hepatic encephalopathy, pyelonephritis, [1, 2] ureter obstruction which can lead to kidney fibrosis [3], etc. Among various responsible factors for colonization of *H. Pylori* presence of urease within bacteria has been found to be most important virulence factor as it causes buffering of stomach acid, produce toxic effect on epithelium cells of stomach, cause disruption of junction of cells tight junctions and sheaths antigens [4–7]. Furthermore it spreads its virulence by catalyzing urea present in human and animal body into ammonia which causes elevation in pH of gastric media and thereby make comfortable environment for the various pathogenic bacteria containing ureases such as *H. pylori* to survive there and spread colonies [8–17].

These infections need attention and proper treatment which can be possible only by inhibiting the responsible enzyme; this compelled us to investigate on urease inhibitors. Drugs used clinically for the treatment of ailments caused by urease producing bacteria includes bismuth complex, phosphoramidates, imidazole derivatives, Antibiotics [18, 19], proton-pump inhibitors [20, 21], H_2_-blockers [22, 23] and hydroxamic acids with double and triple therapy to eradicate the *H. pylori* infection [24, 25]. But cost of therapy, antibiotic resistance, and associated side effects like teratogenic effects shown by hydroxamic acid and rapid disintegration of phosphoramidates at low pH [26–28] motivated researchers towards natural compounds.

A number of biologically active compounds from natural products have been studied against many diseases [29, 30]. One of the most abundant polyphenol present in human being diet specially consumed in green coffee, fruits and vegetables; chlorogenic acid is a naturally occurring ester of quinic acid and caffeic acid [31]. The name chlorogenic acid was introduced in 1846 in response to explain the major component present in beans of green coffee (Payen 1846) [32]. Chlorogenic acid has antioxidant, antimicrobial, and anxiolytic activity antioxidant, antimicrobial, antibacterial, antiviral and anti-inflammatory capacities, which can contribute in the prevention of chronic and cardiovascular diseases, as described by several studies in-vivo and in-vitro [31, 33–40]. Recent reports indicate that this compound also exerts anti-proliferative effects against some types of cancer cell, such as those from liver, blood, or brain cancer [41–43]. Chlorogenic acids not only play a role as antioxidant of coffee, but also contribute to the aroma and taste profile [44, 45]. In pharmaceutical industries it has been in continuous demand because of its beneficial effects [46].

According to literature studies a large number of medicinal plants having phenolic compound and polyphenols as active component have been reported for their anti- *H. Pylori* activity. Recent findings of many researchers stressed that phenolic compounds having strong antioxidant profile play important role in inhibiting *H. pylori.* Literature uncovers the antioxidant and *H. Pylori* urease inhibition profile of chlorogenic acid extracted from Oregano, Cranberry juices as well as extracts of other phytochemical extracts probably by inhibiting urease enzyme [47]. Konstantinopoulou et al. studied phenolic compound chlorogenic acid extracted from plant *Anthemis altissima* against *H. Pylori* bacterium and found that it was quite effective in inhibiting the growth of bacteria with MIC value 312.5–1250 μg/mL [48]. In an application for patent for cosmetic and dermatological composition for anti-rash also used chlorogenic acid extracted from aqueous green coffee extract as anti-urease agent [49]. Shang et al. reported that extract of *L. japonica* containing chlorogenic acid and luteolin glycoside as main component showed good therapeutic potential against *H. Pylori* [50]. Paun et al. reported urease inhibitory activities of extracts of *Geranium* spp.*, Helleborus* spp. and *Hyssopus* spp. polyphenolic extracts having chlorogenic acid as active compound [51]. Furthermore the docking interactions with target protein help in optimization of lead molecules [52] which further help in designing the ligands as potential urease inhibitors. In this regards, targeting urease for treating pathogenic disorders caused by it may open a new line of treatment for infections caused by urease-producing bacteria. This research was conducted in search for newer pharmacophore with emphasis towards urease.

## Results and discussion

### Chemistry

**Scheme 1:**
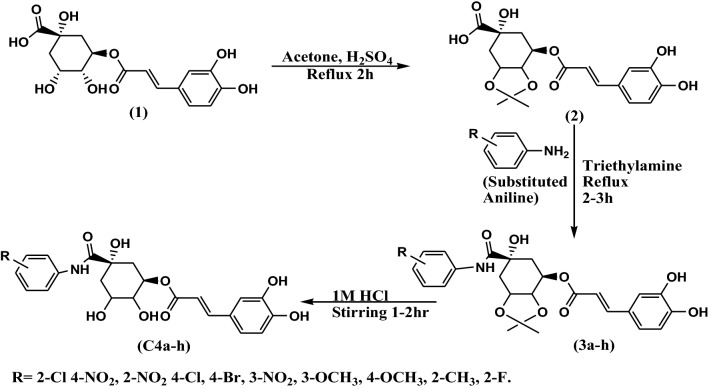
Synthetic scheme forn the synthesis of chlorogenic acid derivatives

Chlorogenic acid derivatives **C4**(**a**–**h**) were synthesized by following the procedure given in Scheme 1 [53–55]. Evaluation of structure of novel derivatives was confirmed by spectroscopic methods such as IR, ^1^H NMR, ^13^CNMR and elemental analysis. Firstly protection of hydroxyl groups of quinic part of chlorogenic acid was done by lactonisation by stirring it with conc. sulphuric acid in acetone for 2 h followed by dilution with ethylacetate and washing with water and brine solution. Anilides of chlorogenic acid was prepared in the second step by refluxing with substituted anilines (Table 1) in presence of dichloromethane for 2–3 h. Finally deprotection of hydroxyl groups of chlorogenic acid was done by reaction with 0.1 M HCl with subsequent washing with ethylacetate in vaccum under pressure. Monitoring of reaction was done by thin layer chromatography and completion of reaction was confirmed by single spot in TLC under UV lamp. Evaluation of structure of novel derivatives was confirmed by spectroscopic methods such as IR, ^1^H NMR, ^13^CNMR, elemental analysis. Derivatisation was confirmed by disappearance of peak of saturated acid (C=O) at 1716 cm^−1^ of chlorogenic acid and appearance of C=O amide peak at 1629–1683 cm^−1^ in compounds **C4**(**a**–**h**). Moreover peak of N–H amide at 3310–3334 cm^−1^ in compounds **C4**(**a**–**h**) also confirmed formation of anilides. ^1^HNMR signals were interpreted by their value of chemical shift for particular protons of synthesized derivatives, coupling constant and multiplicities of signals. Singlet observed at δ 8.5–9.0 was due to NH of secondary amide in all derivatives and in ^13^C NMR signal of carboxylic acid carbon at δ 180 ppm was shifted to 175 ppm due to formation of amide derivative confirmed formation of compound. Further and final confirmation process involves analyzing their mass spectrum for determination of molecular weight in which Q-ToF Micro instrument was used as ion source. Maximum number of the derivatives showed peak at M^+^ (molecular ion peak), (M^++1^), (M^++2^) in positive chemical ionization and (M^+1^), (M^+2^), M^+^ during negative chemical ionization mode. Elemental analysis of chlorogenic acid derivative was carried out in CHNS analyzer where C, H and N in percent were found within acceptable range.

**Table 1:**
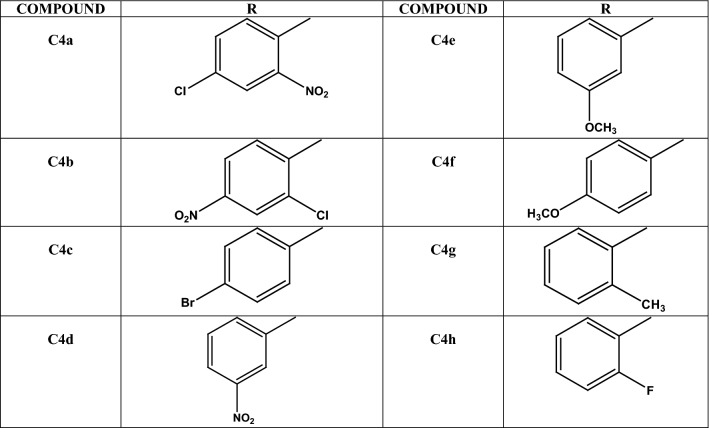
Substituent for the design of chlorogenic acid derivatives (**C4a**–**h**)

### Biological activity

**Fig. 1:**
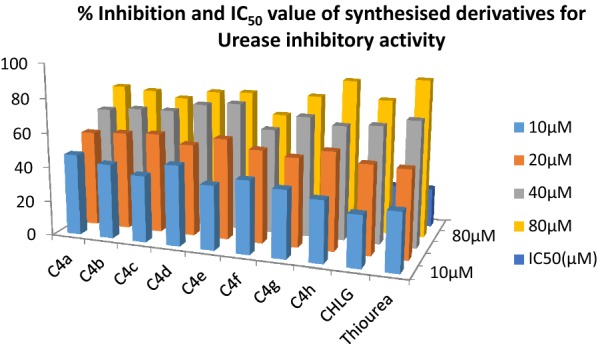
Percentage Inhibition and IC _50_ value of synthesized derivatives for urease inhibitory activity

**Fig. 2:**
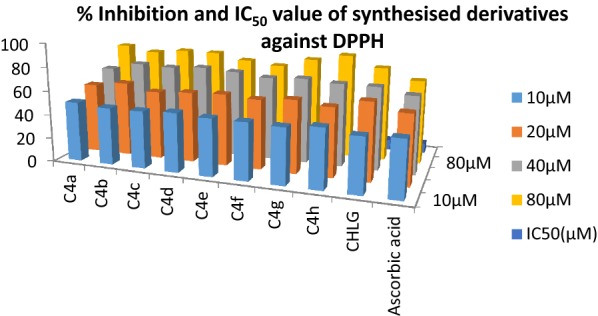
Percentage Inhibition and IC _50_ value of synthesized derivatives for antioxidant activity

It has been recommended that phenolic moiety has been found to be important for antioxidant and urease inhibition property of synthesized compounds. The newly synthesized derivatives of chlorogenic acid were examined for their urease enzyme inhibition, free radical scavenging activity and for *H. Pylori* inhibition. As structure of urease from different sources has common structural features therefore urease inhibition studies were carried out on jack bean urease. Inhibition studies were carried out by indophenol technique described by Weatherburn [56] in which measurement of concentration of ammonia released during the reaction between reagents and compounds were done. Antioxidant property of derivatives was observed by their ability to donate hydrogen or electrons to DPPH [57–59]. According to our investigational data almost all the synthesized derivatives showed good potential in both activities than parent drug chlorogenic acid as well as standard drug having IC_50_ value in range of 11.01–22.02 µM and 5.10–8.48 µM against urease inhibition with competitive mode of inhibition and free radical scavenging activity using thiourea and ascorbic acid as standard drugs respectively (Table 2). According to experimental values derivative **C4a**, **C4d, C4a** and **C4b** bearing nitro and chloro at 2nd and 4th position and **C4d** bearing only nitro group at 3rd position) were found to possess excellent IC_50_ value i.e. 11.01 ± 0.013, 13.8 ± 0.041 and 15.86 ± 0.004 µM respectively in urease inhibition (Fig. 1) and 5.10 ± 0.018, 5.34 ± 0.007 and 6.01 ± 0.005 µM in antioxidant property (Fig. 2) against DPPH. It was observed from the experimental data that substitution of electron withdrawing halogen specially chloro and nitro at anilide ring specifically 2nd and 4th position strengthen the potential of candidate towards enzyme inhibition while electron donating groups like methoxy and methyl groups at 3rd and 4 position (**C4e**, **C4f** and **C4g**) does not showed remarkable potential. Antioxidant activity was also highly influenced by addition of electron withdrawing groups in compound **C4a** and **C4b** (IC_50_ 5.10 ± 0.018 and 6.01 ± 0.005 µM) chloro and nitro group work synergistically and improves hydrogen releasing capacity of phenolic group of parent chlorogenic acid.

**Table 2:** IC_50_ value synthesized compounds for urease inhibition and antioxidant activity

Compound	IC_50_ (µM) urease inhibition^a^	IC_50_ (µM) antioxidant activity^a^
**C4a**	11.01 ± 0.013	5.10 ± 0.018
**C4b**	15.86 ± 0.004	6.01 ± 0.005
**C4c**	22.02 ± 0.027	8.48 ± 0.026
**C4d**	13.8 ± 0.041	5.34 ± 0.007
**C4e**	20.6 ± 0.001	6.85 ± 0.020
**C4f**	21.83 ± 0.009	7.89 ± 0.006
**C4g**	21.5 ± 0.032	7.75 ± 0.011
**C4h**	21.24 ± 0.016	7.23 ± 0.008
CHLG	22.68 ± 0.006	8.55 ± 0.01
Thiourea	22.80 ± 0.011	–
Ascorbic acid	–	8.59 ± 0.004

^a^Values related for the evaluated compound absorption which provide 50% inhibition of Urease inhibition action, and are the mean SEM; statistical significance: p < 0.05 against the equivalent IC_50_ values achieved against urease, as identified through ANOVA/Dunnett’s test

Derivatives **C4a**, **C4b** and **C4d** which were found best in urease inhibition and antioxidant activity as well as in terms of docking score were tested further against antibacterial efficiency against *Helicobacter pylori* DSM 4867 using AHA as a standard and DMSO as control. MIC_50_ values, lowest concentration of compound inhibiting microbial growth after 24 h on agar plates in comparison with positive control [60–63], were calculated for compounds and the results revealed that **C4a** displayed its excellent potency with good zone of inhibition i.e. 10.00 ± 0.00 mm and MIC value 500 μg/mL as compared to standard drug acetohydroxamic acid having zone of inhibition 9.00 ± 0.50 mm and MIC 1000 μg/mL. In-vitro results concluded that compound **C4a** bearing nitro and chloro group at 2nd and 4th position indicated excellent antibacterial action against *H. Pylori* probably by interacting with the target protein efficiently and inhibited the growth of pathogenic bacteria.

### Enzyme kinetics

**Fig. 3:**
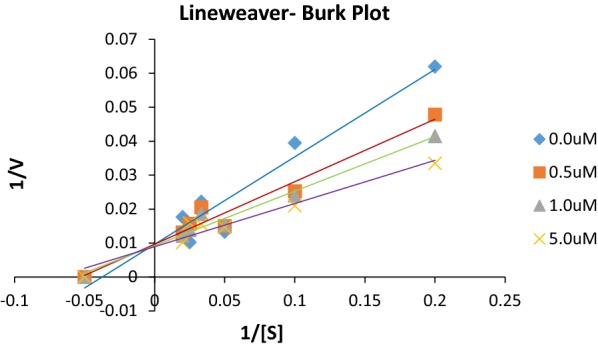
Lineweaver-burk plot for compound C4a

Study of inhibitory effect of chlorogenic derivatives on jack bean urease was performed to check the inhibitory potential, kinetics studies and mechanism of inhibition in phosphate buffer and 1 mM EDTA at pH 8.2. Lineweaver–Burk plots (1/absorbance versus 1/urea) was constructed from kinetic data to determine the mechanism of enzyme inhibition by varying the concentration of substrate urea in the absence and presence of different concentrations of most potent compound **C4a** (Fig. 3). Inhibition constant (Ki) was determined as the intersection on x-axis of the plot of 1/V_max_ and varying concentration of inhibitor obtained from Lineweaver–Burke plot and all experiments were performed in triplicate. Linear lines were formed which intersected at y-axis indicated competitive type of Inhibition as Km was different but V_max_ was same at different concentrations of compound. Competitive inhibition infers that compound **C4a** will probably compete for binding at the site of substrate in enzyme active site pocket. Binding confirmations from molecular simulation studies also confirm the mode of inhibition as shown in Fig. 3.

### Structure activity relationship

**Fig. 4:**
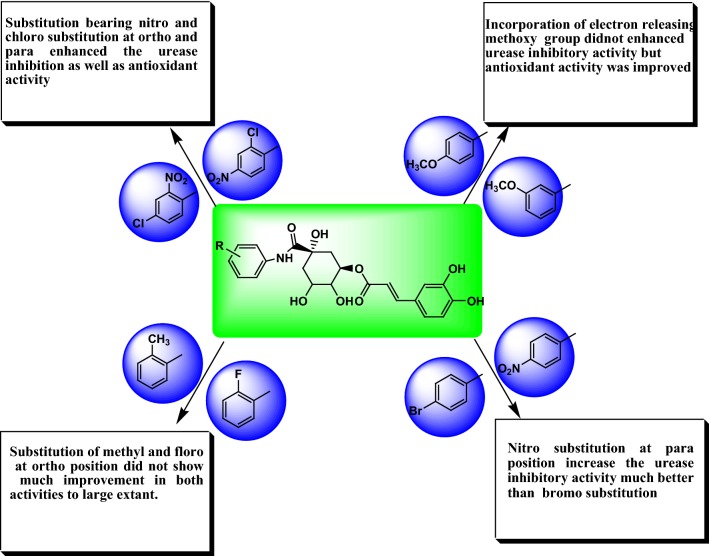
Structure activity relationship of synthesized derivative of chlorogenic acid

Studying results from antioxidant nature and urease inhibition activities of newly synthesized morin derivatives, structure activity relationship can be derived (Fig. 4).

1. Incorporation of aromatic ring bearing chloro and nitro group ortho and para position improved urease inhibition and hydrogen donating capacity of compound (compound **C4a**, **C4b** and **C4d** bearing nitro at 3rd position). Nitro and chloro group probably worked synergistically and make the ligands suitable for proper fitting into the active site of protein thereby increased urease inhibition property, also predicted by molecular docking studies (Fig. 5).

**Fig. 5:**
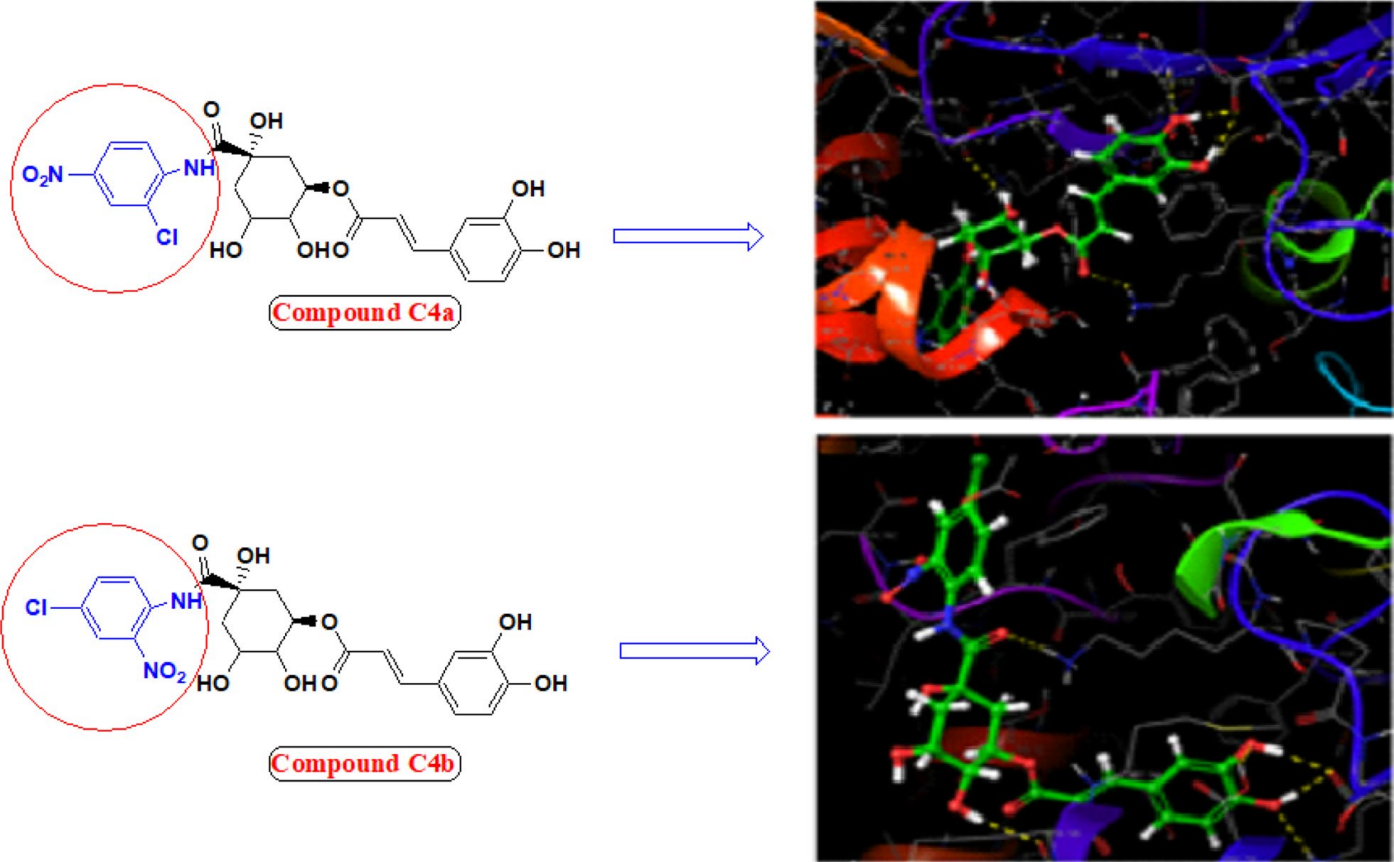
Molecular structure and binding model of most potent compounds **C4a** and **C4b** (Ball and stick coloured by element) within active pocket of jack bean urease 3LA4
2. Presence of Nitro group (**C4a**, **C4b** and **C4d**) improved biological profile of compound.
3. Addition of aromatic ring bearing electron withdrawing groups (**C4e**, **g**) did not much improve the urease efficiency of compound might be by stabilizing the molecule.
4. Incorporation of aromatic ring having bromo and fluoro substituents only improved the antioxidant nature of parent compound (**C4c**, **C4h**).

### Molecular docking study

**Fig. 6:**
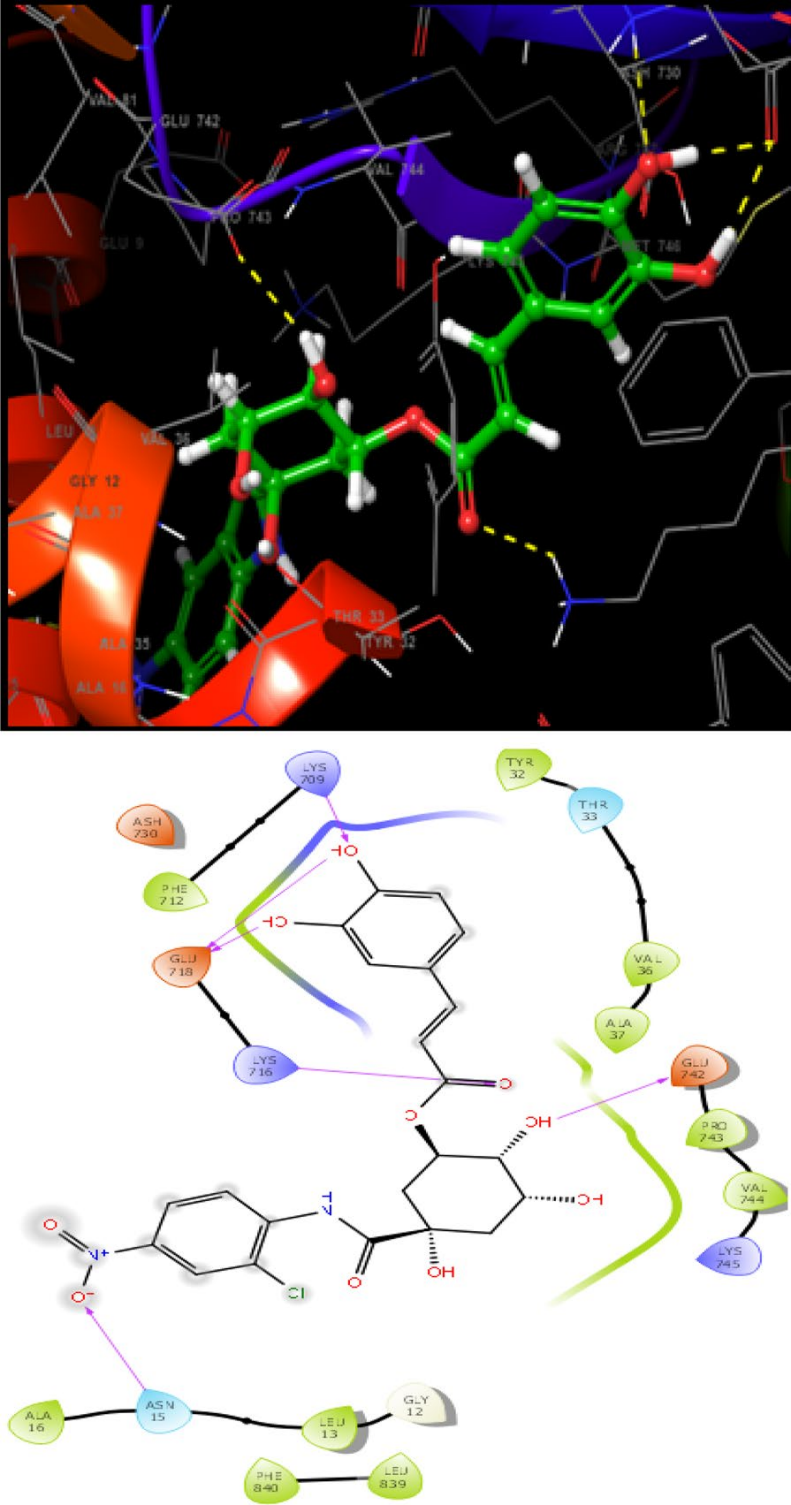
Molecular docking of most potent compound **C4a** in active site of JBU 3LA4 (In 3D binding model ligands are depicted as green sticks whereas interacting amino acid as grey color and hydrogen bond as yellow dotted lines)

Molecular docking is an informative tool for investigating affinity between ligand and protein target. With the aim to insight the binding interaction of the compounds with jack bean urease protein [64–66], Schrodinger’s maestro package of molecular docking software was used for in-silico studies of newly designed library of chlorogenic acid proposed derivatives. Plant urease jack bean having PDB ID 3LA4 having good resolution about 2.0 Å, crystallographic complex was selected for docking the ligands by induced fit docking (IFD) method. Docking parameters revealed that designed ligands possess excellent docking score and binding energy as compared to standard drugs thiourea and AHA (Table 3). Exhaustive ligand protein interaction studies showed that newly synthesized ligands bind within active site of enzyme firmly by hydrogen bond formation, pi–pi stacking and hydrophobic interaction (Table 4). Position and alignment of particular substituents on molecules was found to be responsible for perfect binding of ligand with enzyme. The docking pose of most active ligand (**C4a**) showed astonishing interactions among protein complex with five hydrogen bonds between hydroxyl, carbonyl and nitro group of ligand with residues Lys 709, Glu 718, Lys 716, Glu 742, Asn 15 and possessed excellent docking score and binding energy (− 10.091, − 62.674 kcal/mol). Hydrophobic interactions among ligand and residues Phe 712, Tyr 32, Val 36, Ala 37, Pro 743, Val 744, Ala 16, Leu 13, Leu 839, Phe 840 was noticed which firmly hold the molecule within compact substrate cavity (Fig. 6). Polar residues were held as the supporter to anilide bond of the compound C4a. After assessment of best docking pose of second most potent compound (**C4d**) it was found to form seven hydrogen bonds among Gly 641, Glu 418, Tyr 32, Ser 421, Lys 716, Met 746 with hydroxyl, carboxyl and NH groups. Salt bridge formation was also observed between oxygen and nitrogen atom with Glu 418, Glu 642 residue as shown in Table 4 which additionally provided support to ligand in active site cavity. Hydrogen bond shown by yellow coloured and compounds with green colour with substituents of different coloured. A hydrophobic interaction was also emerged between ligand and surrounding residues Val 640, Pro 717, Tyr 32, Val 36, Phe 712, Val 744, Met 746, Phe 838. Similarly six hydrogen bond formations was observed in inhibitor of third rank (**C4b**) between Lys 716, Glu 718, Ash 730, Lys 709, Phe 840, Val 844 residues and hydroxyl and keto group of ligand. Docking studies revealed that hydrogen bond interactions fix the ligands firmly and tightly in the active site.

**Table 3:** Docking parameters of this studied targets

Compound	Docking score	Binding energy (Kcal/mol)	Glide hbond	Glide evdw	Glide ecoul
**C4a**	− 10.091	− 62.674	− 3.305	− 45.684	− 16.99
**C4b**	− 9.833	− 56.267	− 3.216	− 38.903	− 17.364
**C4c**	− 9.116	− 52.149	− 3.184	− 38.257	− 13.892
**C4d**	− 10.603	− 63.352	− 3.675	− 40.113	− 23.239
**C4e**	− 9.723	− 48.832	− 3.839	− 31.028	− 17.804
**C4f**	− 9.475	− 61.373	− 3.216	− 40.351	− 21.021
**C4g**	− 9.211	− 51.97	− 3.315	− 35.28	− 16.69
**C4h**	− 9.653	− 58.22	− 3.352	− 42.567	− 15.653
Thiourea	− 3.459	− 21.156	− 1.484	− 13.004	− 8.152
AHA	− 3.049	− 17.454	− 1.311	− 8.936	− 8.523

**Table 4:**
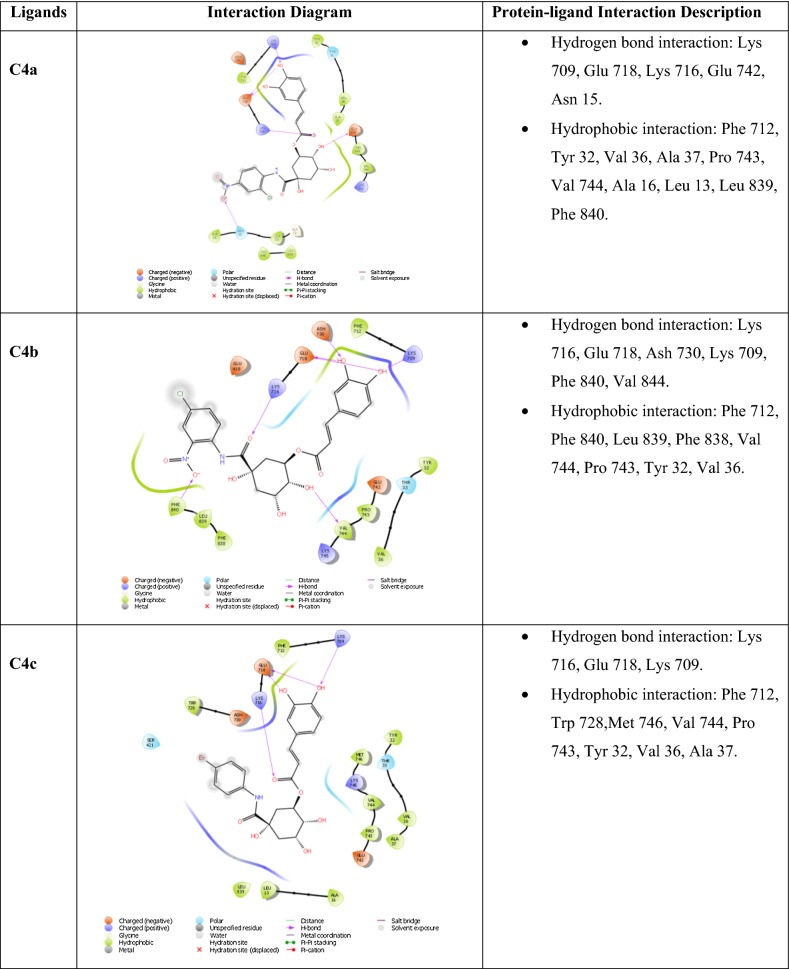

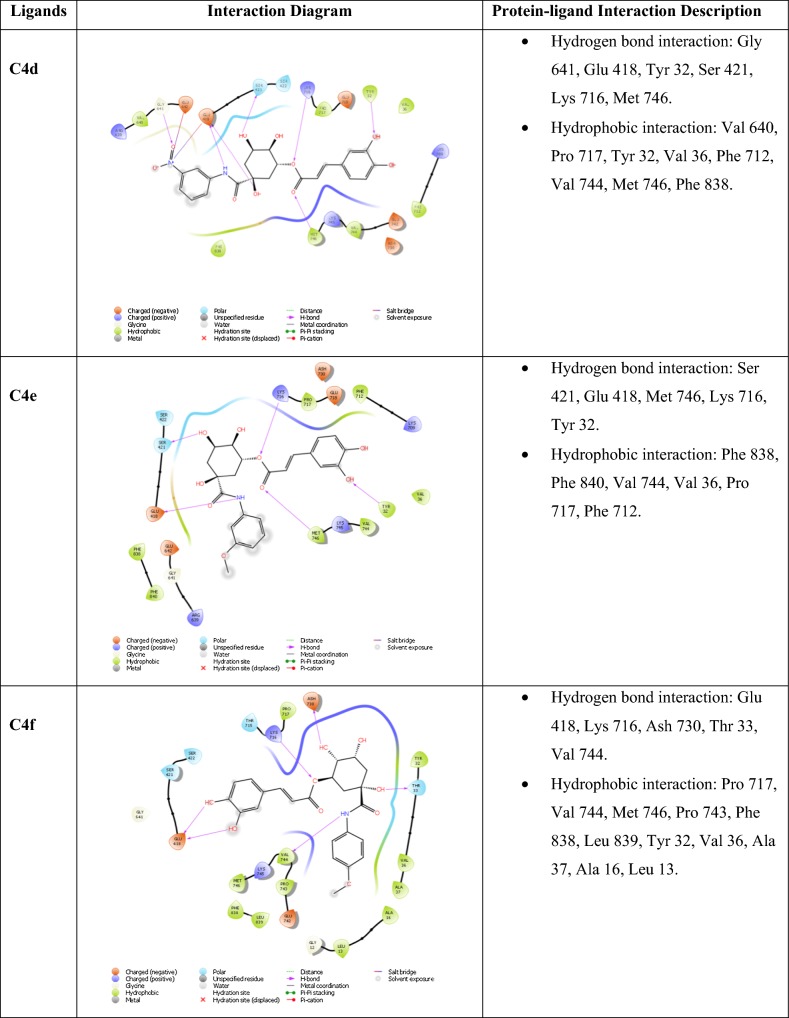

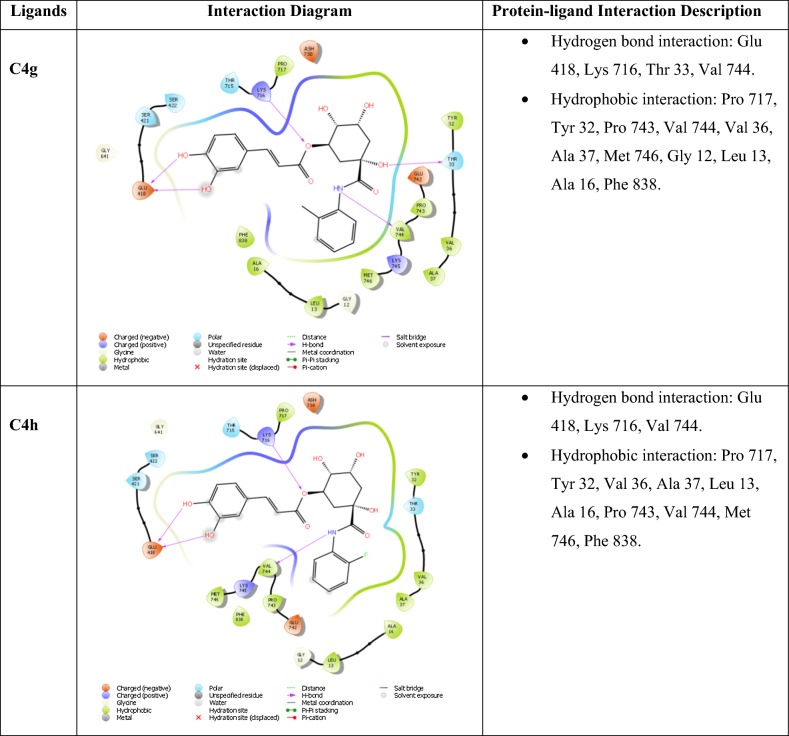
Ligand protein interaction studies

Molecular simulation studies revealed that almost all of the tested inhibitors occupied the same core of enzyme having surrounding residues Lys 709, Glu 718, Lys 716, Glu 742, Asn 15, Phe 712, Tyr 32, Val 36, Ala 37, Pro 743, Val 744, Ala 16, Leu 13, Leu 839 and Phe 840 and every inhibitor showed good hydrogen and hydrophobic interactions. Hydrogen bond formations were considered most important for proper binding of ligand within the enzyme. Almost every molecule have good docking score from − 9.211 to − 10.091 as compared to − 3.459 and − 3.049 of standard thiourea and Acetohydroxamic acid as well as excellent binding energy ranges from − 48.832 kJ/mol to − 63.352 kJ/mol as compared to − 21.156 kJ/mol and − 17.454 kJ/mol of standard thiourea and Acetohydroxamic acid. Docking studies concluded that designed ligands have excellent affinity and binding capability for protein as compared to parent and standard compounds.

### Admet studies

In drug discovering the ADME profile of drug like molecules is very important and for this purpose Schrodinger’s maestro molecular modeling package Qikprop module was utilized. The Absorption, distribution, metabolism and excretion descriptors of the designed molecules are given in Table 5. Blood brain barrier partition coefficient (QPlogBB), estimated IC_50_ value for HERG K^+^ channels obstruction (log HERG), estimation of human serum albumin binding (QPlogKhsa), permeation through skin estimation (QPlogKp), apparent Caco-2 cell permeability estimation in nm/sec (QPPCaco) and apparent MDCK cell permeability estimation in nm/sec (QPPMDCK), partition coefficient in octanol and water Log P, solubility in aqueous media log S, Lipinski’s rule of five and Percent Human Oral Absorption (%HOA). Results revealed that ADME parameters of each ligand within the bounds of satisfactory range without violating Lipinski’s rules [67–70].

**Table 5:** Quikprop simulation studies

Synthesised ligands	QPlogS	QPlog HERG	QPPCaco	QPlogBB	QPPMDCK	QPlogKp	QPlogKhsa	HBD	HBA	LogP o/w	%HOA	Rule of five
**C4a**	− 4.589	− 6.394	4.091	− 4.001	2.616	− 6.062	− 0.4	6	11.15	0.796	45.152	3
**C4b**	− 4.788	− 6.542	6.268	− 3.798	5.074	− 5.658	− 0.392	6	11.15	1.046	47.274	3
**C4c**	− 4.91	− 6.74	24.303	− 3.033	23.635	− 4.48	− 0.338	6	10.15	1.576	35.058	2
**C4d**	− 4.28	− 6.674	2.872	− 4.389	0.885	− 6.231	− 0.478	6	11.15	0.328	37.263	2
**C4e**	− 4.226	− 6.614	26.46	− 3.199	9.758	− 4.345	− 0.451	6	10.9	1.094	45.852	1
**C4f**	− 4.003	− 6.339	26.687	− 3.093	9.848	− 4.383	− 0.439	6	10.9	1.07	45.779	1
**C4g**	− 3.682	− 6.001	40.05	− 2.649	15.273	− 4.055	− 0.362	6	10.15	1.238	49.922	1
**C4h**	− 3.923	− 6.279	33.741	− 2.751	19.076	− 4.178	− 0.429	6	10.15	1.184	48.268	1

In the present study, ADMET calculations of ligands (**C4a**–**h**) showed an acceptable drug-like profile with good availability by oral route and obey rule of five without any considerable violations also many ligands possessed in range values of QPlog S, OPlogHERG, OPPCaco, QPlogBB, QPPMDCK, QPlogKp, QPlogKhsa, HBD, HBA, Log P, % HOA which made them ligands of choice for urease protein

## Experimental

### Materials

Chlorogenic acid was purchased from CDH and Jack bean urease from Sigma Aldrich. Analytical grade reagents and solvents were utilized as a part of study and obtained locally. Progression of reaction was observed via. Thin layer chromatography and recrystallization of products was done in order to purify the compounds which were again checked for purity by thin layer chromatography (TLC) performed on precoated plates (0.25 mm) purchased from Merk and visulisation was done under UV lamp. Measurement of melting point was done on Sonar melting point apparatus in open capillary tubes and was uncorrected. The spectral data, IR and ^1^H NMR, ^13^CNMR were measured by standard procedures. Brucker 12060280, Germany Software: OPUS 7.2.1394 spectrophotometer in cm^−1^ was used for recording IR spectra of derivatives and elemental analysis was done on Perkin–Elmer 2400 C, H, N analyzer. The ^1^HNMR and ^13^CNMR spectra were recorded in DMSO-d_6_ on a Bruker DRX-400 FTNMR instrument. Coupling constants (J) were reported in Hertz (Hz) and chemical shifts were depicted as δ (parts per million). Waters Micromass Q-ToF Micro instrument was used for Mass spectra recording.

### Synthetic procedure

#### General synthetic procedure for preparation of derivatives of chlorogenic acid

##### Synthesis of (1S, 3R)-3-((E)-3-(3,4-dihydroxyphenyl)acryloyloxy)-1-hydroxy-4,5-dimethoxycyclohexane carboxylic acid (**2**)

In a round bottom flask a solution of chlorogenic acid (0.1 g, 0.52 mmol) in acetone (10 mL) was added with addition of conc. sulphuric acid (2 drops) and the reaction mixture was stirred along with refluxing for 2 h. The progress of the reaction was monitored by TLC, after the completion of the reaction, the solvent was allowed to evaporate, 15 mL ethylacetate was added to dilute the mixture and finally washing was done with water (2 × 30 mL) followed by washing with brine solution (20 mL). Finally yielded crude compound 2 (91%) having melting point 175–177 °C.

##### General procedure for synthesis of compounds (**C3a**–**h**)

Amide derivatives of chlorogenic acid was prepared by refluxing a solution of compound 2 (0.1 g, 0.46 mmol) in dichloromethane (5 mL) with substituted aniline (0.7 mmol) at 80 °C for 2–3 h (scheme 1). Reaction progress was continuously monitored with the help of TLC. After the completion of the reaction as indicated by single spot on precoated plate, the solvent was evaporated and diluted with ethyl acetate (25 mL). Mixture was washed with water (250 mL) followed by brine solution (30 mL). The crude product was obtained which was purified by dichloromethane to afford the pure product (**C3a**–**h**).

##### General procedure for synthesis of compounds (**C4a**–**h**)

For deprotecting the hydroxyl groups of synthesized amide derivatives, synthesized compounds C (3a–h) (5.0 g) was dissolved in water (10 mL), then added aq. 1 M HCl (40 mL) at room temperature. The reaction mixture was stirred at room temperature for 1–2 h. Solvent was removed under reduced pressure. The residue HCl was removed by adding EtOEt (20 × 3 mL) under vacuum [53–55]. The residue collected was further dried and product was obtained in good yields.

##### Spectral data

###### (1R, 5S)-5-((2-chloro-4-nitrophenyl) carbamoyl)-2, 3, 5-trihydroxycyclohexyl (**2E**)-3-(3, 4-dihydroxyphenyl)prop-2-enoate (**4a**)

R_f_ TLC mobile phase: Butanol: Water: Acetic acid (6:2:2) = 0.78; (%) Yield = 87.1; m.p (186–188 °C); IR cm^−1^ 3632 (O–H str.), 3328 (N–H str), 3156 (C–H aromatic), 1732 (C=O str ester), 1692 (N–H bending), 1669 (C=O amide), 1636 (C=C str alkene), 1469 (C=C aromatic), 1326 (NO_2_ sym.), 748 (C–Cl); ^1^H NMR (400 MHz, DMSO-*d*_6_) δ 9.40 (s, 2H), 9.32 (s, 1H), 8.73 (s, 1H), 8.32 (d, *J* = 1.5 Hz, 1H), 8.24 (d, *J* = 1.5 Hz, 1H), 8.10 (dd, *J* = 12.4, 7.0 Hz, 1H), 7.47 (d, *J* = 1.6 Hz 1H), 7.04–6.97 (m, 3H), 6.30 (d, *J* = 1.6 Hz, 1H), 5.17 (d, *J* = 4.5 Hz, 1H), 4.98 (s, 1H), 4.78 (td, *J* = 7.1 Hz, 1H), 4.40 (d, *J* = 4.5 Hz 1H), 3.89–3.61 (m, 2H), 2.42 (dt, *J* = 7.1, 1.6 Hz, 2H), 2.24 (dt, *J* = 7.1, 1.6 Hz, 1H), 2.13 (dd, *J* = 12.4, 7.0 Hz, 1H), 2.01 (dd, *J* = 12.4, 7.0 Hz, 1H); ^13^C NMR (100 MHz, CDCl_3_)δ 174.4, 167.2, 147.3, 147.0, 146.2, 140.6, 139.0, 126.1, 120.9, 119.2, 115.6, 77.1, 73.7, 71.1, 69.1, 41.2, 38.1; MS ES + (ToF): m/z 508.09 [M + 1]; CHN: Calc. (C_22_H_21_ClN_2_O_10_): C, 51.93; H, 4.16; N, 5.51. Found: C, 51.92; H, 4.14; N, 5.50.

###### (1R, 5S)-5-((4-chloro-2-nitrophenyl) carbamoyl)-2, 3, 5-trihydroxycyclohexyl (**2E**)-3-(3, 4-dihydroxyphenyl)prop-2-enoate (**4b**)

R_f_ TLC mobile phase: Butanol: Water: Acetic acid (6:2:2) = 0.75; (%) Yield = 79.8; m.p (182–184 °C); IR cm^−1^ 3636 (O–H str.), 3334 (N–H str), 3167 (C–H aromatic), 1739 (C=O str ester), 1691 (N–H bending), 1642 (C=O amide), 1634 (C=C str alkene), 1484 (C=C aromatic), 1328 (NO_2_ sym.), 752 (C–Cl); ^1^H NMR (400 MHz, DMSO-*d*_6_) δ 9.76 (s, 1H), 9.40 (s, 1H), 8.73 (s, 1H), 8.05 (s, 1H), 7.66 (dd, J = 12.1, 1.5 Hz, 1H), 7.54 (dd, *J* = 12.4, 7.0 Hz, 1H), 7.47 (d, *J* = 1.6 Hz, 1H), 7.02 (d, *J* = 4.5 Hz, 1H), 6.95 (dd, *J* = 12.4, 7.0 Hz, 1H), 6.77 (d, *J* = 1.6 Hz, 1H), 6.30 (d, *J* = 1.6 Hz, 1H), 5.00 (s, 1H), 4.90–4.77 (m, 2H), 4.69 (d, J = 15.0 Hz, 1H), 3.82 (p, *J* = 7.0 Hz, 1H), 3.67 (dt, *J* = 11.5 Hz, 1H), 2.31 (dd, *J* = 12.1, 5.0 Hz, 1H), 2.13 (dd, *J* = 12.0, 6.5 Hz, 2H), 1.88 (dd, *J* = 12.1, 1.0 Hz, 1H); ^13^C NMR (100 MHz, CDCl_3_)δ 173.0, 166.2, 148.9, 147.8, 146.8, 136.2, 135.8, 125.5, 125.4, 125.3, 120.3, 116.3, 115.6, 77.7, 71.1, 69.6, 68.9, 41.2, 38.1; MS ES + (ToF): m/z 508.09 [M + 1]; CHN: Calc. (C_22_H_21_ClN_2_O_10_): C, 51.93; H, 4.16; N, 5.51. Found: C, 51.91; H, 4.17; N, 5.52.

###### *(1R, 5S)*-5-((4-bromophenyl)carbamoyl)-2, 3, 5-trihydroxycyclohexyl (**2E**)-3-(3, 4-dihydroxyphenyl)prop-2-enoate (**4c**)

R_f_ TLC mobile phase: Butanol: Water: Acetic acid (6:2:2) = 0.69; (%) Yield = 76.5; m.p (190–192 °C); IR cm^−1^ 3614 (O–H str.), 3310 (N–H str), 3142 (C–H aromatic), 1727 (C=O str ester), 1690 (N–H bending), 1637 (C=O amide), 1629 (C=C str alkene), 1477 (C=C aromatic), 518 (C–Br); ^1^H NMR (400 MHz, DMSO-*d*_6_) δ 9.41 (s, 1H), 8.74 (s, 1H), 8.40 (s, 1H), 7.57–7.43 (m, 5H), 7.04 (d, *J* = 4.5 Hz, 1H), 6.94 (dd, *J* = 12.4, 7.0 Hz, 1H), 6.75 (d, *J* = 1.6 Hz, 1H), 6.29 (d, *J* = 1.6 Hz, 1H), 4.97 (s, 1H), 4.82–4.71 (m, 2H), 4.39 (d, *J* = 4.8 Hz, 1H), 4.25–4.15 (m, 1H), 3.60 (p, *J* = 7.0 Hz, 1H), 2.34 (dd, *J* = 12.4, 7.0 Hz, 1H), 2.24 (dd, *J* = 12.4, 7.0 Hz, 1H), 2.15 (dd, *J* = 12.4, 7.0 Hz 1H), 1.95 (dd, *J* = 12.4, 7.0 Hz,1H); ^13^C NMR (100 MHz, CDCl_3_)δ 175.4, 166.9, 149.1, 147.3, 146.7, 146.4, 146.3, 138.2, 132.0, 128.0, 122.4, 122.1, 117.9, 116.0, 115.9, 115.2, 77.3, 73.1, 71.3, 69.0, 41.5, 38.1; MS ES + (ToF): m/z 507.11 [M + 1]; CHN: Calc. (C_22_H_22_BrNO_8_): C, 51.98; H, 4.36; N, 2.76. Found: C, 51.96; H, 4.38; N, 2.75.

###### (1R, 5S)-2, 3, 5-trihydroxy-5-((3-nitrophenyl)carbamoyl)cyclohexyl (**2E**)-3-(3, 4-dihydroxyphenyl)prop-2-enoate (**4d**)

R_f_ TLC mobile phase: Butanol: Water: Acetic acid (6:2:2) = 0.72; (%) Yield = 71.7; m.p (174–176 °C); IR cm^−1^ 3628 (O–H str.), 3330 (N–H str), 3098 (C–H aromatic), 1736 (C=O str ester), 1639 (C=O amide), 1636 (C=C str), 1477 (C=C aromatic), 1326 (NO_2_ sym.); ^1^H NMR (400 MHz, DMSO-*d*_6_) δ 9.42 (s, 1H), 8.84 (s, 1H), 8.72 (s, 1H), 8.49 (s, 1H), 7.91 (dd, *J* = 12.4, 7.0 Hz, 2H), 7.53 (t, *J* = 1.6 Hz, 1H), 7.46 (d, *J* = 4.8 Hz, 1H), 7.04 (d, *J* = 4.8 Hz, 1H), 6.96 (dd, *J* = 12.4, 7.0 Hz, 1H), 6.77 (d, *J* = 1.6 Hz, 1H), 6.29 (d, *J* = 1.6 Hz, 1H), 5.66 (d, *J* = 1.6 Hz, 1H), 5.45 (s, 1H), 4.80 (s, 1H), 4.51 (td, *J* = 7.5, 4.5 Hz 1H), 3.96–3.71 (m, 2H), 2.30–2.20 (m, 2H), 2.07–2.00 (m, 2H); ^13^C NMR (100 MHz, CDCl_3_)δ 174.2, 166.9, 148.9, 148.6, 148.3, 147.3, 146.4, 139.0, 138.9, 138.5, 131.2, 127.4, 125.9, 122.8, 118.4–117.8, 116.6, 115.7, 115.0, 114.7, 114.5, 114.3, 114.2, 77.3, 73.8, 71.6, 69.0, 41.3, 38.4; MS ES + (ToF): m/z 474.14 [M + 1]; CHN: Calc. (C_22_H_22_BrNO_8_): C, 55.70; H, 4.67; N, 5.90. Found: C, 55.73; H, 4.65; N, 5.93.

###### (1R, 5S)-2, 3, 5-trihydroxy-5-((3-methoxyphenyl)carbamoyl) cyclohexyl (**2E**)-3-(3, 4-dihydroxyphenyl)prop-2-enoate (**4e**)

R_f_ TLC mobile phase: Butanol: Water: Acetic acid (6:2:2) = 0.77; (%) Yield = 83.6; m.p (162–164 °C); IR cm^−1^ 3603 (O–H str.), 3321 (N–H str), 3173 (C–H aromatic), 1745 (C=O str ester), 1658 (C=O amide), 1625 (C=C str), 1475 (C=C aromatic), 1134 (C-O str. alkoxy); ^1^H NMR (400 MHz, DMSO-*d*_6_) δ 9.41 (s, 1H), 8.75 (s, 1H), 8.17 (s, 1H), 7.47 (d, *J* = 1.6 Hz, 1H), 7.37 (t, *J* = 1.6 Hz, 1H), 7.27–7.17 (m, 2H), 7.06 (d, *J* = 1.6 Hz, 1H), 6.95 (dd, *J* = 12.4, 7.0 Hz, 1H), 6.79 (d, *J* = 1.6 Hz 1H), 6.69 (d, *J* = 1.6 Hz 1H), 6.30 (d, *J* = 1.6 Hz 1H), 5.50(t, *J* = 1.6 Hz, 1H), 5.17 (td, *J* = 7.1, 1.6 Hz, 1H), 4.99 (s, 1H), 4.54 (s, 1H), 3.97 (d, *J* = 1.6 Hz, 1H), 3.78 (s, 3H), 3.74–3.69 (m, 1H), 2.39 (dd, *J* = 12.4, 7.0 Hz, 1H), 2.07 (dd, *J* = 12.4, 7.0 Hz, 2H), 1.78 (dd, *J* = 12.4, 7.0 Hz, 1H); ^13^C NMR (100 MHz, CDCl_3_)δ 175.5, 166.8, 160.7, 148.5, 147.0, 146.0, 139.4, 139.2, 129.6, 127.2, 122.7, 120.4, 119.6, 118.4, 117.8, 116.5, 115.7, 115.1, 114.7, 114.5, 114.2, 77.4, 73.9, 71.6, 69.0, 41.4, 38.4; MS ES + (ToF): m/z 459.15 [M + 1]; CHN: Calc. (C_23_H_25_NO_9_): C, 60.13; H, 5.48; N, 3.05; Found: C, 60.12; H, 5.50; N, 3.03.

###### (1R, 5S)-2, 3, 5-trihydroxy-5-((4-methoxyphenyl)carbamoyl)cyclohexyl (**2E**)-3-(3, 4-dihydroxyphenyl)prop-2-enoate (**4f**)

R_f_ TLC mobile phase: Butanol: Water: Acetic acid (6:2:2) = 0.81; (%) Yield = 85.4; m.p (160–162 °C); IR cm^−1^ 3611 (O–H str.), 3325 (N–H str), 3194 (C–H aromatic), 1741 (C=O str ester), 1683 (C=O amide), 1627 (C=C str), 1456 (C=C aromatic), 1145 (C-O str. alkoxy); ^1^H NMR (400 MHz, DMSO-*d*_6_) δ 9.40 (s, 1H), 8.73 (s, 1H), 8.30 (s, 1H), 7.60–7.52 (m, 2H), 7.04 (d, *J* = 1.6 Hz, 1H), 6.99–6.89 (m, 3H), 6.77 (d, *J* = 1.6 Hz 1H), 6.30 (d, *J* = 1.6 Hz, 1H), 4.97 (s, 1H), 4.82–4.91 (m, 2H), 4.39 (d, *J* = 4.5 Hz 1H), 4.26–4.15 (m, 1H), 3.79 (s, 3H), 3.60 (p, 1H), 2.34 (dd, *J* = 7.5, 5.0 Hz, 1H), 2.24 (dd, *J* = 7.5, 5.0 Hz, 1H), 2.16 (dd *J* = 7.5, 5.0 Hz, 1H), 1.97 (dd, 1H); ^13^C NMR (100 MHz, CDCl_3_)δ 175.6, 166.8, 157.0, 148.5, 147.0, 146.1, 134.1, 127.3, 122.7, 122.4, 116.3, 115.6, 115.0, 114.7, 114.5, 114.3, 114.1, 77.2, 73.7, 71.1, 69.0, 41.3, 38.4; MS ES + (ToF): m/z 459.22 [M + 1]; CHN: Calc. (C_23_H_25_NO_9_): C, 60.13; H, 5.48; N, 3.05; Found: C, 60.15; H, 5.47; N, 3.07.

###### (1R, 5S)-2, 3, 5-trihydroxy-5-((2-methylphenyl)carbamoyl)cyclohexyl (**2E**)-3-(3, 4-dihydroxyphenyl)prop-2-enoate (**4g**)

R_f_ TLC mobile phase: Butanol: Water: Acetic acid (6:2:2) = 0.66; (%) Yield = 73.9; m.p (166–168 °C); IR cm^−1^ 3611 (O–H str.), 3325 (N–H str), 3194 (C–H aromatic), 1741 (C=O str ester), 1683 (C=O amide), 1627 (C=C str), 1456 (C=C aromatic), 1145 (C-O str. alkoxy); ^1^H NMR (400 MHz, DMSO-*d*_6_) δ 9.42 (s, 1H), 8.74 (s, 1H), 8.41 (s, 1H), 7.95 (dd, dd, *J* = 7.5, 5.0 Hz, 1H), 7.51–7.37 (m, 2H), 7.27–7.15 (m, 1H), 7.02 (d, *J* = 1.6 Hz, 1H), 6.95 (dd, dd, *J* = 7.5, 5.0 Hz, 1H), 6.78 (d, *J* = 1.6 Hz, 1H), 6.32 (d, *J* = 1.6 Hz, 1H), 4.98 (s, 1H), 4.79 (td, 1H), 4.50 (dd, dd, *J* = 7.5, 5.0 Hz, 2H), 4.24–4.14 (m, H), 3.62 (p, 1H), 2.43 (dd, dd, *J* = 7.5, 5.0 Hz, 1H), 2.33–2.21 (m, 4H), 1.98 (ddd, dd, *J* = 7.5, 5.0, 2.3 Hz 2H); ^13^C NMR (100 MHz, CDCl_3_)δ 175.8, 166.8, 148.5, 147.1, 147.0, 146.9, 146.1, 136.1, 132.5, 132.3, 132.1, 129.9, 127.3, 126.9, 125.0, 122.8, 121.7, 116.7, 115.7, 115.2, 77.1, 73.8, 71.1, 69.0, 41.3, 38.1, 17.7; MS ES + (ToF): m/z 443.16 [M + 1]; CHN: Calc. (C_23_H_25_NO_8_): C, 62.30; H, 5.68; N, 3.16; Found: C, 62.28; H, 5.69; N, 3.14.

###### (1R, 5S)-5-((2-fluorophenyl)carbamoyl)-2, 3, 5-trihydroxycyclohexyl (**2E**)-3-(3, 4-dihydroxyphenyl)prop-2-enoate (**4h**)

R_f_ TLC mobile phase: Butanol: Water: Acetic acid (6:2:2) = 0.71; (%) Yield = 74.2; m.p (178–180 °C); ^1^H NMR (400 MHz, DMSO-*d*_6_) δ 9.44 (s, 1H), 8.70 (s, 1H), 8.36 (s, 1H), 8.02 (dd, *J* = 7.5, 5.0 Hz, 1H), 7.47 (d, *J* = 1.6 Hz, 1H), 7.16 (dd, *J* = 7.4, 1.6 Hz, 2H), 7.07–6.95 (m, 3H), 6.79 (d, *J* = 1.6 Hz, 1H), 6.30 (dd, *J* = 7.4, 1.6 Hz, 1H), 4.97 (s, 1H), 4.82–4.72 (m, 2H), 4.39 (d, *J* = 1.6 Hz, 1H), 4.20 (dt, *J* = 7.1, 1.6 Hz, 1H), 3.60 (p, 1H), 2.35 (dd, *J* = 7.4, 1.6 Hz, 1H), 2.25–2.15 (dd, *J* = 7.4, 1.6 Hz, 2H), 1.99 (dd, *J* = 7.4, 1.6 Hz, H); ^13^C NMR (100 MHz, CDCl_3_)δ 175.6, 166.8, 156.3, 154.3, 148.5, 147.0, 146.0, 127.2, 126.9, 126. 8, 126.7, 126.3, 125.8, 124.9, 122.8, 122.0, 116.6, 116.4, 116.1, 116.0, 115.7, 115.1, 77.1, 73.8, 71.2, 69.2, 69.0, 68.9, 68.8, 41.3, 38.1; MS ES + (ToF): m/z 447 [M + 1]; CHN: Calc.(C_23_H_25_NO_8_): C, 59.06; H, 4.96; N, 3.13; Found: C, 59.06; H, 4.96; N, 3.13.

175.63, 166.84, 156.36, 154.35, 148.55, 147.02, 146.09, 127.29, 126.93–126.65, 126.65–124.85, 122.76, 122.03, 116.56–116.00, 115.70, 115.09, 77.13, 73.79, 71.16, 69.16–68.80, 41.36, 38.11.

### Protocol of in-silico study

*In*-*silico* studies of the designed derivatives of chlorogenic acid were done by Induced fit docking protocol of Schrodinger’s Maestro molecular modeling software. Urease from plant source Jack bean having resolution 2.05 Å (PDB code: 3LA4) was retrieved from protein databank online (http://www.rcsb.org) from the Research Collaboratory for Structural bioinformatics (RCSB) and has been used as for the purpose of docking the newly designed ligands in X-ray crystallized structure form for finding the active lead compound.

#### Protein preparation

With the help of protein preparation wizard named prepwiz, protein was prepared for docking studies. In this process assigning bond orders, partial charges and hydrogens were added, side chains and loops having missing atoms were fabricated, all waters (with exception of those which were coordinated to metals) and unnecessary atoms were expelled and alternate conformations were performed.

#### Generation of grid

After preparation of protein area for docking interaction with ligands was selected on protein by forming a grid over it. Grid generation was done by grid generation module of Schrodinger Maestro programming according to the conventions.

#### Ligand preparation

Proposed structure of ligand were drawn by Chemdraw Ultra 8.0 (ChemOffice Package) software which were finally saved as mol. file format and finally prepared by LigPrep module. Addition or removal of hydrogen bond were done for ideal confirmation of ligands, partial charges were computed according to the OPLS-2005 force field with 32 stereo isomers, tautomers, and other options like ionization at the physiological pH 7 ± 2 were set as default options. Ligands were selected for simulation studies after their preparation based upon their minimum potential energy. Geometry of ligand was minimized by application of force field algorithm according to protocol of software.

#### Molecular docking

Ligand-receptor interaction study using maestro package was done according to the given protocol. While performing Grid-based Ligand Docking ligands were allocated at various positions of grid within range of 1 Å and were rotated in all three angles and glide score (*GScore* Grid-based Ligand Docking with Energetics) scoring function was obtained as the output for each ligand given in Table 2. Derivatives possessing higher score in terms of glide score and those possessing lower binding energy were selected for further synthesis process [65–70].

### Urease inhibitory assay

Urease enzyme inhibition investigation studies for all synthesized compounds were done by the method developed by Weatherburn (1967) named Indophenol against Jack Bean Urease [56]. Briefly, incubation of solution of Jack bean protein 25 μL was done at temperature of 30 °C for a period of 15 min with 55 μL of buffers solution having 100 mM urea and 5 μL of test solution in 96-well plates. 45 μL phenol solution composing phenol 1% w/v and solution of sodium nitroprusside 0.005% w/v and alkali reagent 70 μL composed of sodium hydroxide 0.5% w/v and active sodium hypochloride solution 0.1% were mixed to every well. The measurement of increase in absorption at 625 nm was done after 50 min by help of a microplate reader (Molecular Device, USA). Readings were recorded in triplicate set in a final volume of 200 μL using thiourea as standard. Each assay was performed at pH 8.2 (0.01 M K_2_HPO_4_.3H_2_O, 0.01 M LiCl_2_ and 1 mM EDTA). Calculation of Inhibition in percentage of synthesized derivatives was done by the formula given as:

$${\text{I\% }} = \frac{{{\text{A}}_{\text{Control}} - {\text{A}}_{\text{Sample}}}}{{{\text{A}}_{\text{Control}}}} \times 100$$

where, A_control_ is control absorbance; A_sample_ is test absorbance.

### Dpph assay for antioxidant activity

Antioxidant nature of the synthesized derivatives was measured by the help of DPPH method. According to this method synthesized compounds were allowed to react for 0.5 h at 37 °C with stable free radical, 1, 1-diphenyl-2-picrylhydrazyl radical (DPPH). The DPPH was taken in a concentration of 300 µM. Decrease in value of absorption at 515 nm was measured after the period of incubation using a microplate reader using ascorbic acid as a standard. Molecule capable of donating proton to DPPH and cause its reduction acts as an antioxidant. Following reduction deep violet coloured DPPH solution changes to yellow depending upon the nature of antioxidant compound, which brings a measurable decrease in value of absorption at 517 nm [57–59]. The proton or electron donating capacity of the derivative was estimated from the fading of deep purple colored methanolic solution of 1, 1-diphenylpicrylhydrazyl (DPPH). Incubation of mixture was done at room temperature for 30 min and measurements were done at 517 nm against blank. Calculation of percentage inhibition was done by following formula:

$${\text{I\% }} = \frac{{{\text{A}}_{\text{Control}} - {\text{A}}_{\text{Sample}}}}{{{\text{A}}_{\text{Control}}}} \times 100$$

where, A_control_ is control absorbance; A_sample_ is test absorbance.

### Anti- *H. pylori* activity

Since *H. Pylori* is not acidophilic in nature therefore it requires urease, which produced ammonia by hydrolyzing urea present in stomach, for its survival in acidic gastric medium. Because of virulence nature of urease researchers decided to target its inhibition for anti-*H. Pylori* drugs. Novel synthesized derivatives were investigated against *H. pylori* bacterium (DSM 4867, AHA as standard) for their antibacterial activity by using Well diffusion technique. The stock solution of compounds in DMSO (1000 μg/mL) were prepared. Cell suspension was prepared from culture grown on BHI broth. The cell suspensions of all the cultures were adjusted to 1–2 × 10^5^ cells/mL. *Helicobacter pylori* (100 µL) was inoculated by spread plate technique on agar plates (90 mm). Agar wells (5 mm) were made on *H. pylori* inoculated media and impregnated with 2500 µg of each sample and standard which were incubated @ 35 °C for 24–48 h with 5% CO_2_ and observed for zone of inhibition around the well. MIC was determined against *Helicobacter pylori* by micro broth dilution technique as per NCCLS method [60–63].

### Kinetic studies

Kinetic studies of the derivatives were done to calculate the rate constant Vmax and Michaelis constant Km by plotting Lineweaver bruke plot of 1/[S] (substrate concentration) against 1/[V] velocity. To examine the mode of inhibition compound **C4a** was solubilized in DMSO and further dilutions were prepared accordingly. Plot was prepared by using varying concentration of substrate (urea) in with varying concentration of inhibitor **C4a**. Inhibitory constant values (Ki) were determined as the interaction on the X-axis of the plots of the slope against different concentration of derivatives **C4a**. Mode of inhibition was found to be competitive as value of Km was different but V_max_ was same. All experiments were performed in triplicate and kinetic study for interaction of enzyme with inhibitor was computed with Graphpad prism 7.0 software.

### Statistical analysis

Output of statistical analysis has been depicted in mean ± SEM whereas statistical examination of data collected experimentally was done by one-way analysis of variance (ANOVA). Considerable difference revealed by ANOVA p < 0.05 was regarded significant. Evaluation of statistical data was done by Graph Pad Prism 7.0 Version for Windows (San Diego, CA, USA).

## Conclusion

In conclusion, selected derivatives of chlorogenic acid were synthesized after in silico screening of library of designed derivatives against jack bean urease enzyme. Computational studies helped in selection of potential candidate also shed light into the underlying mechanism of inhibition. Synthetic procedure includes three step reactions which involve lactone formation of chlorogenic acid followed by reaction with substituted anilines. Novel synthesized derivatives were evaluated for their urease inhibitory, DPPH- free radical scavenging and Anti- *H. Pylori* activity by in vitro method. Among the series all molecules were found effective and specific in their action with good IC_50_ values and MIC against *H. Pylori*. Designed candidate **C4a** was found top most in all parameters and hence can be the potential lead compound in future for treatment of pathologies caused by urease as well as against *H. Pylori* infection.

## Abbreviations

ADMET: absorption distribution metabolism excretion
MIC: Minimum Inhibitory Concentration
HCl: hydrochloric acid
TLC: thin layer chromatography
UV: ultra violet
DPPH: 2, 2-diphenyl-1-picrylhydrazyl
CHLG: chlorogenic acid
SEM: Standard Error Mean
ANOVA: analysis of variance
DMSO: dimethyl sulphoxide
AHA: acetohydroxamic acid
EDTA: ethylenediaminetetraacetic acid
HOA: Human Oral Absorption
RCSB: Research Collaboratory for Structural bioinformatics
NCCLS: National Committee for Clinical Laboratory Standards
EtOEt: ethylacetoacetate
